# What kind of systematic review should I conduct? A proposed typology and guidance for systematic reviewers in the medical and health sciences

**DOI:** 10.1186/s12874-017-0468-4

**Published:** 2018-01-10

**Authors:** Zachary Munn, Cindy Stern, Edoardo Aromataris, Craig Lockwood, Zoe Jordan

**Affiliations:** The Joanna Briggs Institute, The University of Adelaide, 55 King William Road, North Adelaide, Soueth Australia 5005 Australia

**Keywords:** Systematic reviews, Evidence-based healthcare, Question development

## Abstract

**Background:**

Systematic reviews have been considered as the pillar on which evidence-based healthcare rests. Systematic review methodology has evolved and been modified over the years to accommodate the range of questions that may arise in the health and medical sciences. This paper explores a concept still rarely considered by novice authors and in the literature: determining the type of systematic review to undertake based on a research question or priority.

**Results:**

Within the framework of the evidence-based healthcare paradigm, defining the question and type of systematic review to conduct is a pivotal first step that will guide the rest of the process and has the potential to impact on other aspects of the evidence-based healthcare cycle (evidence generation, transfer and implementation). It is something that novice reviewers (and others not familiar with the range of review types available) need to take account of but frequently overlook. Our aim is to provide a typology of review types and describe key elements that need to be addressed during question development for each type.

**Conclusions:**

In this paper a typology is proposed of various systematic review methodologies. The review types are defined and situated with regard to establishing corresponding questions and inclusion criteria. The ultimate objective is to provide clarified guidance for both novice and experienced reviewers and a unified typology with respect to review types.

## Introduction

Systematic reviews are the gold standard to search for, collate, critique and summarize the best available evidence regarding a clinical question [1, 2]. The results of systematic reviews provide the most valid evidence base to inform the development of trustworthy clinical guidelines (and their recommendations) and clinical decision making [2]. They follow a structured research process that requires rigorous methods to ensure that the results are both reliable and meaningful to end users. Systematic reviews are therefore seen as the pillar of evidence-based healthcare [3–6]. However, systematic review methodology and the language used to express that methodology, has progressed significantly since their appearance in healthcare in the 1970’s and 80’s [7, 8]. The diachronic nature of this evolution has caused, and continues to cause, great confusion for both novice and experienced researchers seeking to synthesise various forms of evidence. Indeed, it has already been argued that the current proliferation of review types is creating challenges for the terminology for describing such reviews [9]. These fundamental issues primarily relate to a) the types of questions being asked and b) the types of evidence used to answer those questions.

Traditionally, systematic reviews have been predominantly conducted to assess the effectiveness of health interventions by critically examining and summarizing the results of randomized controlled trials (RCTs) (using meta-analysis where feasible) [4, 10]. However, health professionals are concerned with questions other than whether an intervention or therapy is effective, and this is reflected in the wide range of research approaches utilized in the health field to generate knowledge for practice. As such, Pearson and colleagues have argued for a pluralistic approach when considering what counts as evidence in health care; suggesting that not all questions can be answered from studies measuring effectiveness alone [4, 11]. As the methods to conduct systematic reviews have evolved and advanced, so too has the thinking around the types of questions we want and need to answer in order to provide the best possible, evidence-based care [4, 11].

Even though most systematic reviews conducted today still focus on questions relating to the effectiveness of medical interventions, many other review types which adhere to the principles and nomenclature of a systematic review have emerged to address the diverse information needs of healthcare professionals and policy makers. This increasing array of systematic review options may be confusing for the novice systematic reviewer, and in our experience as educators, peer reviewers and editors we find that many beginner reviewers struggle to achieve conceptual clarity when planning for a systematic review on an issue other than effectiveness. For example, reviewers regularly try to force their question into the PICO format (population, intervention, comparator and outcome), even though their question may be an issue of diagnostic test accuracy or prognosis; attempting to define all the elements of PICO can confound the remainder of the review process. The aim of this article is to propose a typology of systematic review types aligned to review questions to assist and guide the novice systematic reviewer and editors, peer-reviewers and policy makers. To our knowledge, this is the first classification of types of systematic reviews foci conducted in the medical and health sciences into one central typology.

### Review typology

For the purpose of this typology a systematic review is defined as a robust, reproducible, structured critical synthesis of existing research. While other approaches to the synthesis of evidence exist (including but not limited to literature reviews, evidence maps, rapid reviews, integrative reviews, scoping and umbrella reviews), this paper seeks only to include approaches that subscribe to the above definition. As such, ten different types of systematic review foci are listed below and in Table 1. In this proposed typology, we provide the key elements for formulating a question for each of the 10 review types.

1. Effectiveness reviews [12]
2. Experiential (Qualitative) reviews [13]
3. Costs/Economic Evaluation reviews [14]
4. Prevalence and/or Incidence reviews [15]
5. Diagnostic Test Accuracy reviews [16]
6. Etiology and/or Risk reviews [17]
7. Expert opinion/policy reviews [18]
8. Psychometric reviews [19]
9. Prognostic reviews [20]
10. Methodological systematic reviews [21, 22]

**Table 1:** Types of reviews

Review Type	Aim	Question Format	Question Example
Effectiveness	To evaluate the effectiveness of a certain treatment/practice in terms of its impact on outcomes	Population, Intervention, Comparator/s, Outcomes (PICO) [23]	What is the effectiveness of exercise for treating depression in adults compared to no treatment or a comparison treatment? [69]
Experiential (Qualitative)	To investigate the experience or meaningfulness of a particular phenomenon	Population, Phenomena of Interest, Context (PICo) [13]	What is the experience of undergoing high technology medical imaging (such as Magnetic Resonance Imaging) in adult patients in high income countries? [70]
Costs/Economic Evaluation	To determine the costs associated with a particular approach/treatment strategy, particularly in terms of cost effectiveness or benefit	Population, Intervention, Comparator/s, Outcomes, Context (PICOC) [14]	What is the cost effectiveness of self-monitoring of blood glucose in type 2 diabetes mellitus in high income countries? [71]
Prevalence and/or Incidence	To determine the prevalence and/or incidence of a certain condition	Condition, Context, Population (CoCoPop) [15]	What is the prevalence/incidence of claustrophobia and claustrophobic reactions in adult patients undergoing MRI? [72]
Diagnostic Test Accuracy	To determine how well a diagnostic test works in terms of its sensitivity and specificity for a particular diagnosis	Population, Index Test, Reference Test, Diagnosis of Interest (PIRD) [16]	What is the diagnostic test accuracy of nutritional tools (such as the Malnutrition Screening Tool) compared to the Patient Generated Subjective Global Assessment amongst patients with colorectal cancer to identify undernutrition? [73]
Etiology and/or Risk	To determine the association between particular exposures/risk factors and outcomes	Population, Exposure, Outcome (PEO) [17]	Are adults exposed to radon at risk for developing lung cancer? [74]
Expert opinion/policy	To review and synthesize current expert opinion, text or policy on a certain phenomena	Population, Intervention or Phenomena of Interest, Context (PICo) [18]	What are the policy strategies to reduce maternal mortality in pregnant and birthing women in Cambodia, Thailand, Malaysia and Sri Lanka? [75]
Psychometric	To evaluate the psychometric properties of a certain test, normally to determine how the reliability and validity of a particular test or assessment.	Construct of interest or the name of the measurement instrument(s), Population, Type of measurement instrument, Measurement properties [31, 32]	What is the reliability, validity, responsiveness and interpretability of methods (manual muscle testing, isokinetic dynamometry, hand held dynamometry) to assess muscle strength in adults? [76]
Prognostic	To determine the overall prognosis for a condition, the link between specific prognostic factors and an outcome and/or prognostic/prediction models and prognostic tests.	Population, Prognostic Factors (or models of interest), Outcome (PFO) [20, 34–36]	In adults with low back pain, what is the association between individual recovery expectations and disability outcomes? [77]
Methodology	To examine and investigate current research methods and potentially their impact on research quality.	Types of Studies, Types of Data, Types of Methods, Outcomes [39] (SDMO)	What is the effect of masked (blind) peer review for quantitative studies in terms of the study quality as reported in published reports? (question modified from Jefferson 2007) [40]

### Effectiveness reviews

Systematic reviews assessing the effectiveness of an intervention or therapy are by far the most common. Essentially effectiveness is the extent to which an intervention, when used appropriately, achieves the intended effect [11]. The PICO approach (see Table 1) to question development is well known [23] and comprehensive guidance for these types of reviews is available [24]. Characteristics regarding the population (e.g. demographic and socioeconomic factors and setting), intervention (e.g. variations in dosage/intensity, delivery mode, and frequency/duration/timing of delivery), comparator (active or passive) and outcomes (primary and secondary including benefits and harms, how outcomes will be measured including the timing of measurement) need to be carefully considered and appropriately justified.

### Experiential (qualitative) reviews

Experiential (qualitative) reviews focus on analyzing human experiences and cultural and social phenomena. Reviews including qualitative evidence may focus on the engagement between the participant and the intervention, as such a qualitative review may describe an intervention, but its question focuses on the perspective of the individuals experiencing it as part of a larger phenomenon. They can be important in exploring and explaining why interventions are or are not effective from a person-centered perspective. Similarly, this type of review can explain and explore why an intervention is not adopted in spite of evidence of its effectiveness [4, 13, 25]. They are important in providing information on the patient’s experience, which can enable the health professional to better understand and interact with patients. The mnemonic PICo can be used to guide question development (see Table 1). With qualitative evidence there is no outcome or comparator to be considered. A phenomenon of interest is the experience, event or process occurring that is under study, such as response to pain or coping with breast cancer; it differs from an intervention in its focus. Context will vary depending on the objective of the review; it may include consideration of cultural factors such as geographic location, specific racial or gender based interests, and details about the setting such as acute care, primary healthcare, or the community [4, 13, 25]. Reviews assessing the experience of a phenomenon may opt to use a mixed methods approach and also include quantitative data, such as that from surveys. There are reporting guidelines available for qualitative reviews, including the ‘Enhancing transparency in reporting the synthesis of qualitative research’ (ENTREQ) statement [26] and the newly proposed meta-ethnography reporting guidelines (eMERGe) [27].

### Costs/economic evaluation reviews

Costs/Economics reviews assess the costs of a certain intervention, process, or procedure. In any society, resources available (including dollars) have alternative uses. In order to make the best decisions about alternative courses of action evidence is needed on the health benefits and also on the types and amount of resources needed for these courses of action. Health economic evaluations are particularly useful to inform health policy decisions attempting to achieve equality in healthcare provision to all members of society and are commonly used to justify the existence and development of health services, new health technologies and also, clinical guideline development [14]. Issues of cost and resource use may be standalone reviews or components of effectiveness reviews [28]. Cost/Economic evaluations are examples of a quantitative review and as such can follow the PICO mnemonic (see Table 1). Consideration should be given to whether the entire world/international population is to be considered or only a population (or sub-population) of a particular country. Details of the intervention and comparator should include the nature of services/care delivered, time period of delivery, dosage/intensity, co-interventions, and personnel undertaking delivery. Consider if outcomes will only focus on resource usage and costs of the intervention and its comparator(s) or additionally on cost-effectiveness. Context (including perspective) can also be considered in these types of questions e.g. health setting(s).

### Prevalence and/or incidence reviews

Essentially prevalence or incidence reviews measure disease burden (whether at a local, national or global level). Prevalence refers to the proportion of a population who have a certain disease whereas incidence relates to how often a disease occurs. These types of reviews enable governments, policy makers, health professionals and the general population to inform the development and delivery of health services and evaluate changes and trends in diseases over time [15, 29]. Prevalence or incidence reviews are important in the description of geographical distribution of a variable and the variation between subgroups (such as gender or socioeconomic status), and for informing health care planning and resource allocation. The CoCoPop framework can be used for reviews addressing a question relevant to prevalence or incidence (see Table 1). Condition refers to the variable of interest and can be a health condition, disease, symptom, event of factor. Information regarding how the condition will be measured, diagnosed or confirmed should be provided. Environmental factors can have a substantial impact on the prevalence or incidence of a condition so it is important that authors define the context or specific setting relevant to their review question [15, 29]. The population or study subjects should be clearly defined and described in detail.

### Diagnostic test accuracy reviews

Systematic reviews assessing diagnostic test accuracy provide a summary of test performance and are important for clinicians and other healthcare practitioners in order to determine the accuracy of the diagnostic tests they use or are considering using [16]. Diagnostic tests are used by clinicians to identify the presence or absence of a condition in a patient for the purpose of developing an appropriate treatment plan. Often there are several tests available for diagnosis. The mnemonic PIRD is recommended for question development for these types of systematic reviews (see Table 1). The population is all participants who will undergo the diagnostic test while the index test(s) is the diagnostic test whose accuracy is being investigated in the review. Consider if multiple iterations of a test exist and who carries out or interprets the test, the conditions the test is conducted under and specific details regarding how the test will be conducted. The reference test is the ‘gold standard’ test to which the results of the index test will be compared. It should be the best test currently available for the diagnosis of the condition of interest. Diagnosis of interest relates to what diagnosis is being investigated in the systematic review. This may be a disease, injury, disability or any other pathological condition [16].

### Etiology and/or risk reviews

Systematic reviews of etiology and risk are important for informing healthcare planning and resource allocation, and are particularly valuable for decision makers when making decisions regarding health policy and prevention of adverse health outcomes. The common objective of many of these types of reviews is to determine whether and to what degree a relationship exists between an exposure and a health outcome. Use of the PEO mnemonic is recommended (see Table 1). The review question should outline the exposure, disease, symptom or health condition of interest, the population or groups at risk, as well as the context/location, the time period and the length of time where relevant [17]. The exposure of interest refers to a particular risk factor or several risk factors associated with a disease/condition of interest in a population, group or cohort who have been exposed to them. It should be clearly reported what the exposure or risk factor is, and how it may be measured/identified including the dose and nature of exposure and the duration of exposure, if relevant. Important outcomes of interest relevant to the health issue and important to key stakeholders (e.g. knowledge users, consumers, policy makers, payers etc.) must be specified. Guidance now exists for conducting these types of reviews [17]. As these reviews rely heavily on observational studies, the Meta-analysis Of Observational Studies in Epidemiology (MOOSE) [30] reporting guidelines should be referred to in addition to the PRISMA guidelines.

### Expert opinion/policy reviews

Expert opinion and policy analysis systematic reviews focus on the synthesis of narrative text and/or policy. Expert opinion has a role to play in evidence-based healthcare, as it can be used to either complement empirical evidence or, in the absence of research studies, stand alone as the best available evidence. The synthesis of findings from expert opinion within the systematic review process is not well recognized in mainstream evidence-based practice. However, in the absence of research studies, the use of a transparent systematic process to identify the best available evidence drawn from text and opinion can provide practical guidance to practitioners and policy makers [18]. While a number of mnemonics have been discussed previously that can be used for opinion and text, not all elements necessarily apply to every text or opinion-based review, and use of mnemonics should be considered a guide rather than a policy. Broadly PICo can be used where I can refer to either the intervention or a phenomena of interest (see Table 1). Reviewers will need to describe the population, giving attention to whether specific characteristics of interest, such as age, gender, level of education or professional qualification are important to the question. As with other types of reviews, interventions may be broad areas of practice management, or specific, singular interventions. However, reviews of text or opinion may also reflect an interest in opinions around power, politics or other aspects of health care other than direct interventions, in which case, these should be described in detail. The use of a comparator and specific outcome statement is not necessarily required for a review of text and opinion based literature. In circumstances where they are considered appropriate, the nature and characteristics of the comparator and outcomes should be described [18].

### Psychometric reviews

Psychometric systematic reviews (or systematic reviews of measurement properties) are conducted to assess the quality/characteristics of health measurement instruments to determine the best tool for use (in terms of its validity, reliability, responsiveness etc.) in practice for a certain condition or factor [31–33]. A psychometric systematic review may be undertaken on a) the measurement properties of one measurement instrument, b) the measurement properties of the most commonly utilized measurement instruments measuring a specific construct, c) the measurement properties of all available measurement instruments to measure a specific construct in a specific population or d) the measurement properties of all available measurement instruments in a specific population that does not specify the construct to be measured. The COnsensus-based Standards for the selection of health Measurement Instruments (COSMIN) group have developed guidance for conducting these types of reviews [19, 31]. They recommend firstly defining the type of review to be conducted as well as the construct or the name(s) of the outcome measurement instrument(s) of interest, the target population, the type of measurement instrument of interest (e.g. questionnaires, imaging tests) and the measurement properties on which the review investigates (see Table 1).

### Prognostic reviews

Prognostic research is of high value as it provides clinicians and patients with information regarding the course of a disease and potential outcomes, in addition to potentially providing useful information to deliver targeted therapy relating to specific prognostic factors [20, 34, 35]. Prognostic reviews are complex and methodology for these types of reviews is still under development, although a Cochrane methods group exists to support this approach [20]. Potential systematic reviewers wishing to conduct a prognostic review may be interested in determining the overall prognosis for a condition, the link between specific prognostic factors and an outcome and/or prognostic/prediction models and prognostic tests [20, 34–37]. Currently there is little information available to guide the development of a well-defined review question however the Quality in Prognosis Studies (QUIPS) tool [34] and the Checklist for critical appraisal and data extraction for systematic reviews of prediction modelling studies (CHARMS Checklist) [38] have been developed to assist in this process (see Table 1).

### Methodology systematic reviews

Systematic reviews can be conducted for methodological purposes [39], and examples of these reviews are available in the Cochrane Database [40, 41] and elsewhere [21]. These reviews can be performed to examine any methodological issues relating to the design, conduct and review of research studies and also evidence syntheses. There is limited guidance for conducting these reviews, although there does exist an appendix in the Cochrane Handbook focusing specifically on methodological reviews [39]. They suggest following the SDMO approach where the types of studies should define all eligible study designs as well as any thresholds for inclusion (e.g. RCTS and quasi-RCTs). Types of data should detail the raw material for the methodology studies (e.g. original research submitted to biomedical journals) and the comparisons of interest should be described under types of methods (e.g. blinded peer review versus unblinded peer review) (see Table 1). Lastly both primary and secondary outcome measures should be listed (e.g. quality of published report) [39].

## Discussion

The need to establish a specific, focussed question that can be utilized to define search terms, inclusion and exclusion criteria and interpretation of data within a systematic review is an ongoing issue [42]. This paper provides an up-to-date typology for systematic reviews which reflects the current state of systematic review conduct. It is now possible that almost any question can be subjected to the process of systematic review. However, it can be daunting and difficult for the novice researcher to determine what type of review they require and how they should conceptualize and phrase their review question, inclusion criteria and the appropriate methods for analysis and synthesis [23]. Ensuring that the review question is well formed is of the utmost importance as question design has the most significant impact on the conduct of a systematic review as the subsequent inclusion criteria are drawn from the question and provide the operational framework for the review [23]. In this proposed typology, we provide the key elements for formulating a question for each of the 10 review types.

When structuring a systematic review question some of these key elements are universally agreed (such as PICO for effectiveness reviews) whilst others are more novel. For example, the use of PIRD for diagnostic reviews contrasts with other mnemonics, such as PITR [43], PPP-ICP-TR [44] or PIRATE [45]. Qualitative reviews have sometimes been guided by the mnemonic SPIDER, however this has been recommended against for guiding searching due to it not identifying papers that are relevant [46]. Variations on our guidance exist, with the additional question elements of ‘time’ (PICOT) and study types (PICOS) also existing. Reviewers are advised to consider these elements when crafting their question to determine if they are relevant for their topic. We believe that based on the guidance included in this typology, constructing a well-built question for a systematic review is a skill that can be mastered even for the novice reviewer.

Related to this discussion of a typology for systematic reviews is the issue of how to distinguish a systematic review from a literature review. When searching the literature, you may come across papers referred to as ‘systematic reviews,’ however, in reality they do not necessarily fit this description [21]. This is of significant concern given the common acceptance of systematic reviews as ‘level 1’ evidence and the best study design to inform practice. However, many of these reviews are simply literature reviews masquerading as the ideal product. It is therefore important to have a critical eye when assessing publications identified as systematic reviews. Today, the methodology of systematic reviews continues to evolve. However, there is general acceptance of certain steps being required in a systematic review of any evidence type [2] and these should be used to distinguish between a literature review and a systematic review. The following can be viewed as the defining features of a systematic review and its conduct [1, 2]:

1. Clearly articulated objectives and questions to be addressed
2. Inclusion and exclusion criteria, stipulated a priori (in a protocol), that determine the eligibility of studies
3. A comprehensive search to identify all relevant studies, both published and unpublished
4. A process of study screening and selection
5. Appraisal of the quality of included studies/ papers (risk of bias) and assessment of the validity of their results/findings/ conclusions
6. Analysis of data extracted from the included research
7. Presentation and synthesis of the results/ findings extracted
8. Interpret the results, potentially establishing the certainty of the results and making and implications for practice and research
9. Transparent reporting of the methodology and methods used to conduct the review

Prior to deciding what type of review to conduct, the reviewer should be clear that a systematic review is the best approach. A systematic review may be undertaken to confirm whether current practice is based on evidence (or not) and to address any uncertainty or variation in practice that may be occurring. Conducting a systematic review also identifies where evidence is not available and can help categorize future research in the area. Most importantly, they are used to produce statements to guide decision-making. Indications for systematic reviews:

1. uncover the international evidence
2. confirm current practice/ address any variation
3. identify areas for future research
4. investigate conflicting results
5. produce statements to guide decision-making

The popularity of systematic reviews has resulted in the creation of various evidence review processes over the last 30 years. These include integrative reviews, scoping reviews [47], evidence maps [48], realist syntheses [49], rapid reviews [50], umbrella reviews (systematic reviews of reviews) [51], mixed methods reviews [52], concept analyses [53] and others. Useful typologies of these diverse review types can be used as reference for researchers, policy makers and funders when discussing a review approach [54, 55]. It was not the purpose of this article to describe and define each of these diverse evidence synthesis methods as our focus was purely on systematic review questions. Depending on the researcher, their question/s and their resources at hand, one of these approaches may be the best fit for answering a particular question.

Gough and colleagues [9] provided clarification between different review designs and methods but stopped short of providing a taxonomy of review types. The rationale for this was that in the field of evidence synthesis ‘the rate of development of new approaches to reviewing is too fast and the overlap of approaches too great for that to be helpful.’ [9] They instead provide a useful description of how reviews may differ and more importantly why this may be the case. It is also our view that evidence synthesis methodology is a rapidly developing field, and that even within the review types classified here (such as effectiveness [56] or experiential [qualitative [57]]) there may be many different subsets and complexities that need to be addressed. Essentially, the classifications listed above may be just the initial level of a much larger family tree. We believe that this typology will provide a useful contribution to efforts to sort and classify evidence review approaches and understand the need for this to be updated over time. A useful next step might be the development of a comprehensive taxonomy to further guide reviewers in making a determination about the most appropriate evidence synthesis product to undertake for a particular purpose or question.

Systematic reviews of animal studies (or preclinical systematic reviews) have not been common practice in the past (when comparing to clinical research) although this is changing [58–61]. Systematic reviews of these types of studies can be useful to inform the design of future experiments (both preclinical and clinical) [59] and address an important gap in translation science [5, 60]. Guidance for these types of reviews is now emerging [58, 60, 62–64]. These review types, which are often hypothesis generating, were excluded from our typology as they are only very rarely used to answer a clinical question.

Systematic reviews are clearly an indispensable component in the chain of scientific enquiry in a much broader sense than simply to inform policy and practice and therefore ensuring that they are designed in a rigorous manner, addressing appropriate questions driven by clinical and policy needs is essential. With the ever-increasing global investment in health research it is imperative that the needs of health service providers and end users are met. It has been suggested that one way to ensure this occurs is to precede any research investment with a systematic review of existing research [65]. However, the only way that such a strategy would be effective would be if all reviews conducted are done so with due rigour.

It has been argued recently that there is mass production of reviews that are often unnecessary, misleading and conflicted with most having weak or insufficient evidence to inform decision making [66]. Indeed, *asking* has been identified as a core functional competency associated with obtaining *and applying* the best available evidence [67]. Fundamental to the tenets of evidence-based healthcare and, in particular evidence implementation, is the ability to formulate a question that is amenable to obtaining evidence and “structured thinking” around question development is critical to its success [67]. The application of evidence can be significantly hampered when existing evidence does not correspond to the situations that practitioners (or guideline developers) are faced with. Hence, determination of appropriate review types that respond to relevant clinical and policy questions is essential.

The revised JBI Model of Evidence-Based Healthcare clarifies the conceptual integration of evidence generation, synthesis, transfer and implementation, “linking how these occur with the necessarily challenging dynamics that contribute to whether translation of evidence into policy and practice is successful” [68]. Fundamental to this approach is the recognition that the process of evidence-based healthcare is not prescriptive or linear, but bi-directional, with each component having the potential to affect what occurs on either side of it. Thus, a systematic review can impact upon the types of primary research that are generated as a result of recommendations produced in the review (evidence generation) but also on the success of their uptake in policy and practice (evidence implementation). It is therefore critical for those undertaking systematic reviews to have a solid understanding of the type of review required to respond to their question.

For novice reviewers, or those unfamiliar with the broad range of review types now available, access to a typology to inform their question development is timely. The typology described above provides a framework that indicates the antecedents and determinants of undertaking a systematic review. There are several factors that may lead an author to conduct a review and these may or may not start with a clearly articulated clinical or policy question. Having a better understanding of the review types available and the questions that these reviews types lend themselves to answering is critical to the success or otherwise of a review. Given the significant resource required to undertake a review this first step is critical as it will impact upon what occurs in both evidence generation and evidence implementation. Thus, enabling novice and experienced reviewers to ensure that they are undertaking the “right” review to respond to a clinical or policy question appropriately has strategic implications from a broader evidence-based healthcare perspective.

## Conclusion

Systematic reviews are the ideal method to rigorously collate, examine and synthesize a body of literature. Systematic review methods now exist for most questions that may arise in healthcare. This article provides a typology for systematic reviewers when deciding on their approach in addition to guidance on structuring their review question. This proposed typology provides the first known attempt to sort and classify systematic review types and their question development frameworks and therefore it can be a useful tool for researchers, policy makers and funders when deciding on an appropriate approach.

## Abbreviations

CHARMS: CHecklist for critical Appraisal and data extraction for systematic Reviews of prediction Modelling Studies
CoCoPop: Condition, Context, Population
COSMIN: COnsensus-based Standards for the selection of health Measurement Instruments
EBHC: Evidence-based healthcare
eMERGe: Meta-ethnography reporting guidelines
ENTREQ: Enhancing transparency in reporting the synthesis of qualitative research
JBI: Joanna Briggs Institute
MOOSE: Meta-analysis Of Observational Studies in Epidemiology
PEO: Population, Exposure, Outcome
PFO: Population, Prognostic Factors (or models of interest), Outcome
PICO: Population, Intervention, Comparator, Outcome
PICo: Population, Phenomena of Interest, Context
PICOC: Population, Intervention, Comparator/s, Outcomes, Context
PIRD: Population, Index Test, Reference Test, Diagnosis of Interest
QUIPS: Quality in Prognosis Studies
RCT: Randomised controlled trial
SDMO: Studies, Data, Methods, Outcomes
